# Global adaptation complicates the interpretation of genome scans for local adaptation

**DOI:** 10.1002/evl3.208

**Published:** 2020-12-15

**Authors:** Tom R. Booker, Sam Yeaman, Michael C. Whitlock

**Affiliations:** ^1^ Department of Forest and Conservation Sciences University of British Columbia Vancouver Canada; ^2^ Biodiversity Research Centre University of British Columbia Vancouver Canada; ^3^ Department of Biological Sciences University of Calgary Calgary Canada; ^4^ Department of Zoology University of British Columbia Vancouver Canada

**Keywords:** *F_ST_* outlier, genetics of adaptation, genome scans, global adaptation, local adaptation

## Abstract

Spatially varying selection promotes variance in allele frequencies, increasing genetic differentiation between the demes of a metapopulation. For that reason, outliers in the genome‐wide distribution of summary statistics measuring genetic differentiation, such as *F_ST_*, are often interpreted as evidence for alleles that contribute to local adaptation. However, theoretical studies have shown that in spatially structured populations the spread of beneficial mutations with spatially uniform fitness effects can also induce transient genetic differentiation. In recent years, numerous empirical studies have suggested that such species‐wide, or global, adaptation makes a substantial contribution to molecular evolution. In this perspective, we discuss how commonly such global adaptation may influence the genome‐wide distribution of *F_ST_* and generate genetic differentiation patterns, which could be mistaken for local adaptation. To illustrate this, we use forward‐in‐time population genetic simulations assuming parameters for the rate and strength of beneficial mutations consistent with estimates from natural populations. We demonstrate that the spread of globally beneficial mutations in parapatric populations may frequently generate *F_ST_* outliers, which could be misinterpreted as evidence for local adaptation. The spread of beneficial mutations causes selective sweeps at flanking sites, so in some cases, the effects of global versus local adaptation may be distinguished by examining patterns of nucleotide diversity within and between populations in addition to *F_ST_*. However, when local adaptation has been only recently established, it may be much more difficult to distinguish from global adaptation, due to less accumulation of linkage disequilibrium at flanking sites. Through our discussion, we conclude that a large fraction of *F_ST_* outliers that are presumed to arise from local adaptation may instead be due to global adaptation.

**Impact summary**

Local adaptation occurs when a population of organisms adapts to the conditions that occur locally and is commonly observed in spatially heterogeneous environments. Finding the genes responsible for such local adaptation is a common goal for both understanding the process of evolution and for managing critical populations. One way to identify the genetic basis of local adaptation is to search for genes that are most different between local populations, using a so‐called *F_ST_* outlier test. In this paper, we show that such patterns of genetic differentiation between local populations may also commonly occur because of adaptation to global conditions, where a single mutation sweeps across the range of a species. This can occur either when populations are observed partway through the sweep process or when recombination during the sweep leaves a spatially variable pattern in the frequency of hitchhiking alleles. The spatial genetic pattern that results from either mechanism is similar to the pattern expected under local adaptation, with high values of *F_ST_*. Although the ways that global adaptation can generate such extreme patterns in *F_ST_* were previously recognized, we show that this global adaptation process likely happens often enough to be a significant source of false positives in searches for the genetic basis of local adaptation.

Determining the genetic basis of local adaptation is of primary interest to evolutionary biologists, as it provides insights into how natural selection shapes genetic variation. The genetic basis of local adaptation has increasingly been studied using various types of “genome scans,” that is, methods which are applied across the genome to detect genetic patterns that are expected under the process of local adaptation (reviewed in Haasl and Payseur 2016; Hoban et al. 2016). Some genome scans look for alleles that are correlated with particular putative selective features of the environment. Others look for genomic regions of particularly high genetic differentiation among populations, based on the fact that local adaptation will, by definition, locally increase the frequency of locally beneficial alleles. As a result, local adaptation should cause some increase in the variation among populations in allele frequencies.

One of the most commonly used summary statistics for such genome scans is Wright's *F_ST_* (or its derivatives), which measures the variance in allele frequencies among the demes of a metapopulation. Because long‐term local adaptation promotes variance in allele frequency among demes, regions of the genome subject to spatially varying selection should have *F_ST_* values that appear extreme in the genome‐wide distribution (Lewontin and Krakauer 1973). Here, we use the term local adaptation to refer to a particular genotype by environment interaction for fitness that could be assessed via common garden experiments; specifically where individuals from a given deme have higher fitness "at home" versus "away," following Kawecki and Ebert (2004). Note that this definition of local adaptation encompasses both alleles that are spatially antagonistic as well as conditionally beneficial. Alleles that have spatially antagonistic effects on fitness act as local barriers to gene flow, which allows *F_ST_* to accumulate over time. Depending on the strength of selection and the rate of migration, spatially varying selection can result in neutral variants linked to selected alleles also exhibiting elevated *F_ST_* (Petry 1983; Bengtsson 1985; Barton and Bengtsson 1986). The population genetic signature of local adaptation due to conditionally beneficial alleles has not received the extensive theoretical treatment that spatial antagonism has. However, simulations have shown that as conditionally beneficial mutations spread through the regions where they are favored, they may cause a transient increase in *F_ST_*, which would dissipate over time (Mee and Yeaman 2019). Because local adaptation is expected to cause extreme values of *F_ST_*, the genes or other functional elements in the genomic regions containing *F_ST_* outliers can provide researchers with a set of hypotheses regarding the loci involved in local adaptation.

However, interpreting the results of genome scans is fraught with difficulties. There are many other reasons aside from local adaptation for why a particular genomic region may have a measure of genetic differentiation that is greater than expected by a particular neutral model. Most genome‐scan methods make implicit assumptions about the pattern of population structure and evolutionary history; violation of these assumptions can result in high false positive rates (Lotterhos and Whitlock 2015). Additionally, neutral sites closely linked to functional loci such as protein‐coding genes or regulatory elements may exhibit reduced diversity due to selective sweeps and/or background selection. Processes that decrease nucleotide diversity can, in some circumstances, cause elevated *F_ST_* if reductions differ within populations versus between populations (Charlesworth 1998; Nachman and Payseur 2012; Cruickshank and Hahn 2014; Zeng and Corcoran 2015, but see Matthey‐Doret and Whitlock 2019). In this perspective, we focus on how frequently selective sweeps caused by the spread of uniformly beneficial mutations generate such *F_ST_* peaks.

In structured populations, selective sweeps may influence the genetic differentiation among populations. When a beneficial allele spreads through a population, the haplotype it is present on will spread too. Once the beneficial allele has fixed across the metapopulation, neutral (or nearly neutral) alleles on the haplotype may also end up at high frequencies. Selective sweeps will, therefore, homogenize genetic variation among populations and typically cause a reduction in nucleotide diversity within and between populations (Barton 2000) and can reduce *F_ST_* about the selected locus (Santiago and Caballero 2005). However, selective sweeps can occasionally generate *F_ST_* outliers by at least two processes. First, as beneficial mutations spread to high frequency in their demes of origin there may be period of lag before they migrate and establish in other demes. If this period of lag is substantial, it may correspond to an ephemeral increase in *F_ST_* if alleles which are rare in one population are driven to high frequency in others (Slatkin and Wiehe 1998; Kim and Maruki 2011; Feder et al. 2019). If a population is sampled during this period of lag, *F_ST_* outliers may be observed in some cases—we refer to *F_ST_* outliers generated by this process as the “lag type.” Second, during a selective sweep, recombination may move beneficial alleles onto different genetic backgrounds in different demes. If different haplotypes are driven to high frequency in different demes, there may be elevated *F_ST_* in regions surrounding selected loci in the wake of a sweep, which is then eroded by subsequent migration (Bierne 2010)—we refer to *F_ST_* outliers generated by this process as the “Bierne type.” Note that a related process can occur in panmictic populations, where it has been referred to as the “soft shoulder” effect (Schrider et al. 2015). It is worth remembering that not every selective sweep in a structured population will generate an *F_ST_* outlier, but occasionally some will. Thus, depending on the frequency of globally beneficial mutations and the selective sweeps that arise from them, global adaptation may often influence genomic patterns of *F_ST_* observed in structured populations.

Although the potential for *F_ST_* outliers to be driven by global adaptation is well recognized, it is unclear how often we should expect this to be a problem for scans using *F_ST_* to detect local adaptation. To answer that question, one may ask how common are such global selective sweeps? The best information on the process of global adaptation in eukaryotes comes from *Drosophila melanogaster*. An estimate of the number of incomplete selective sweeps in that species can be obtained as follows: it is estimated that *D. melanogaster* and *Drosophila simulans* began to diverge around 14 million generations ago (Obbard et al. 2012). In that time, *D. melanogaster* has accumulated 0.0067 substitutions/bp at nonsynonymous sites (Begun et al. 2007). The proportion of nonsynonymous substitutions driven by positive selection (*α*) in *D. melanogaster* has been estimated in numerous studies to be ∼0.5 (Begun et al. 2007; Eyre‐Walker and Keightley 2009; Messer and Petrov 2013; Elyashiv et al. 2016). There are approximately 15 Mbp of nonsynonymous sites in the *D. melanogaster* genome, calculated as two‐thirds of the sites in protein‐coding regions (based on *D. melanogaster* genome build 6.28; downloaded from FlyBase FB2019_03; Thurmond et al. 2019). Taken together, this suggests that an estimated (0.5 × 15 × 0.0067)/14 = 0.0035 adaptive substitutions have occurred at nonsynonymous sites each generation since *D. melanogaster* split with *D. simulans*. Put another way, substitutions of advantageous alleles have occurred approximately every 280 generations in *D. melanogaster*. Thus, if advantageous mutations take longer than 280 generations to fix, multiple sweeps will be going on at any one time. With knowledge of the effective population size (*N_e_*) and selection coefficients for beneficial mutations (*s_a_*), expected fixation times can be calculated (Ewens 1979). It has been estimated that advantageous nonsynonymous mutations in *D. melanogaster* have scaled effects on fitness of *2N_e_s_a_* = 250 (Campos et al. 2017), but note that there is likely a distribution of fitness effects for new beneficial mutations. If we assume that the *N_e_* for *D. melanogaster* has historically been 10^6^, advantageous mutations at nonsynonymous sites in *D. melanogaster* would take ∼97,500 generations to fix and spend ∼22,200 generations at intermediate frequencies (i.e., between 0.2 and 0.8) under panmixia. Taken together, these calculations suggest that at any point in time, *D. melanogaster* is subject to approximately 22,200/280 = 80 incomplete selective sweeps for alleles that will ultimately reach fixation. This calculation assumes a panmictic population, but population structure is ubiquitous in the natural world. Because population structure prolongs fixation times relative to panmixia (Whitlock 2003), 80 incomplete sweeps may be an underestimate. This back‐of‐the‐envelope calculation is obviously quite rough, but nevertheless gives an idea of the possible order of magnitude of the number of ongoing sweeps.

In the above calculation, we assumed that α = 0.5 implies that half of all nonsynonymous substitutions were globally beneficial. Numerous biological processes may occur in spatially extended populations that could conceivably contribute to between‐species divergence at nonsynonymous sites and therefore inflate α, but do not involve global adaptation. For example, mutations that were beneficial in one part of a species’ range but were neutral elsewhere (i.e., conditionally beneficial) may fix due to the force of migration from demes where they are favored. Alternatively, local extinctions could cause alleles that were once spatially restricted in their distribution to fix through the remnants of the species range. We currently do not know how much between‐species divergence is generated by global adaptation or by local adaptation.

Nevertheless, if there are numerous ongoing sweeps in a population, as suggested by the *Drosophila* calculation presented above, some incomplete global sweeps may induce ephemeral genetic differentiation and be detected when scanning the genome for local adaptation (Slatkin and Wiehe 1998; Santiago and Caballero 2005; Bierne 2010; Kim and Maruki 2011; Feder et al. 2019). In the following sections, we describe how frequently globally beneficial mutations may influence the landscape of genetic variability in structured populations and the genomic signatures such adaptation leaves behind. We focus our attention on a model that assumes that the adaptive differences that accumulate between species are solely due to global adaptation, but we remind the reader that, as above, other processes may influence rates of substitution estimated for natural populations.

## ONGOING AND RECENT SELECTIVE SWEEPS OF GLOBALLY BENEFICIAL MUTATIONS CONFOUND AND COMPLICATE THE INTERPRETATION OF GENOME SCANS

To assess how the rate of adaptive substitutions influences the landscape of *F_ST_*, we simulated a pair of parapatric populations experiencing uniformly positive selection but not local adaptation. We then scanned the genome for *F_ST_*. All the *F_ST_* peaks we identified were therefore generated either by global adaptation or genetic drift. We simulated populations of 2*N_e_* individuals divided into two equally sized demes that exchanged an expected *N_e_m* migrants per generation. Simulated chromosomes contained “gene‐like” regions where a proportion, *p_a_*, of mutations was beneficial and had semidominant fitness effects drawn from an exponential distribution with mean ${\bar{S}}_{a}$. Recombination and mutation rates were uniform across the chromosome. Simulations were performed using *SLiM* (v3.2; Haller and Messer 2019). A fuller description of our methods is provided in the Supporting Information Materials.

**Figure 1:**
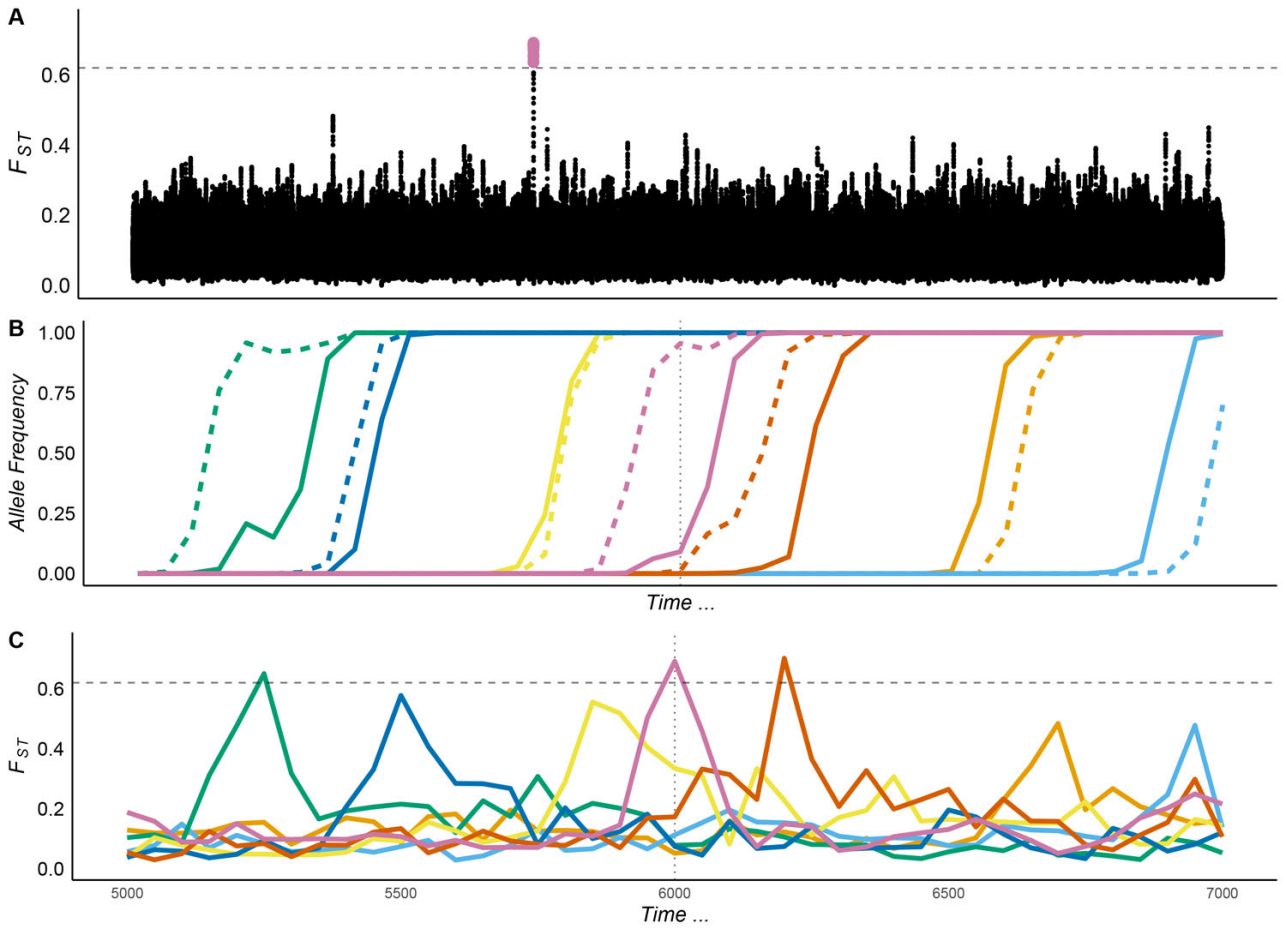
Selective sweeps of globally beneficial alleles can generate ephemeral *F_ST_* peaks. (A) Manhattan plot of *F_ST_* calculated between parapatric populations subject only to global adaptation. *F_ST_* was calculated in sliding windows of 10,000 bp with a step size of 500 bp. (B) The allele frequency of beneficial mutations in deme 1 (solid lines) and deme 2 (dashed lines). (C) *F_ST_* over time for 10,000 bp analysis windows containing beneficial alleles. The vertical red line indicates the time point for which the Manhattan plot in A) was constructed. The dashed gray horizontal line indicates the 99.999th percentile of *F_ST_* from neutral simulations. Simulation parameters, *N_e_* = 2000 diploid individuals, ${\bar{S}}_{a}$ = 0.1, *p_a_* = 0.0001, *Nm* = 1.

The Manhattan plot in Figure 1A shows a typical sliding‐window genome scan for *F_ST_* performed on data from a simulated parapatric population. For the purposes of demonstration, the simulations shown in Figure 1 were performed using *N* = 2000 diploid individuals (1000 individuals in each of two demes). The simulated genome consisted of 500 Mbp of simulated sequence, with 25 Mbp of functional sites. The number of functional sites was chosen to approximately reflect the total number of nonsynonymous sites and sites in the untranslated regions of protein‐coding genes in *D. melanogaster*. Advantageous mutations occurred at functional sites with fitness effects and rates similar to those that have been estimated for *D. melanogaster* (Campos et al. 2017). Importantly, in these simulations no mutations were allowed that had differential fitness in the two populations—all evolution was purely due to drift and globally beneficial alleles.

In the center of the Manhattan plot shown in Figure 1A, there is a clearly defined region of high *F_ST_*, which exceeded the 99.999th percentile of values observed in neutral simulations. If one performed a genome scan on real data and observed an outlier such as the one shown in Figure 1A, it would perhaps be tempting to interpret it as evidence of local adaptation. However, this pattern was caused by the transient differentiation induced by the fixation of a globally beneficial allele. When sampling the same simulated population over time, we observed regions of high *F_ST_* in different genomic locations at some time points and no outliers at others (Fig. S1).

As described in the introduction, there are at least two types of *F_ST_* peaks that can be generated by global adaptation, the “lag type” and the “Bierne type,” and Figure 1B shows instances of both. The pink lines in Figure 1B and C show the change in allele frequency over time for one particular beneficial mutation and the associated *F_ST_*. The beneficial mutation occurs in one deme and spreads to high frequency before it establishes in the other (Figure 1B). During this period of lag, *F_ST_* in the region around the selected site is elevated but drops off once the mutation reaches high frequency in the second deme (Fig. 1C). The Manhattan plot shown in Figure 1A shows the genomic landscape of *F_ST_* during this period of lag, and Figure 1C shows how the central peak dissipated over time. The yellow lines in Figure 1B and C show that elevated *F_ST_* can persist once a beneficial mutation has gone to fixation, and this may occur as a result of the process described by Bierne (2010). For the purpose of visualization, Figure 1B and C show the frequency and associated *F_ST_* profiles for seven beneficial alleles, but many more may be segregating in the population as a whole at any one time generating a heterogeneous landscape of differentiation (Fig. S1).

## *F_ST_* OUTLIERS GENERATED BY GLOBAL ADAPTATION CAN BE COMMON

That global adaptation can influence the genomic landscape of differentiation is clear (Fig. 1; Slatkin and Wiehe 1998; Santiago and Caballero 2005; Bierne 2010; Kim and Maruki 2011), but what is less clear is the frequency with which it does. With a greater number of mutations sweeping to fixation at any one time, there will be a greater chance of observing *F_ST_* peaks driven by global adaptation.

**Figure 2:**
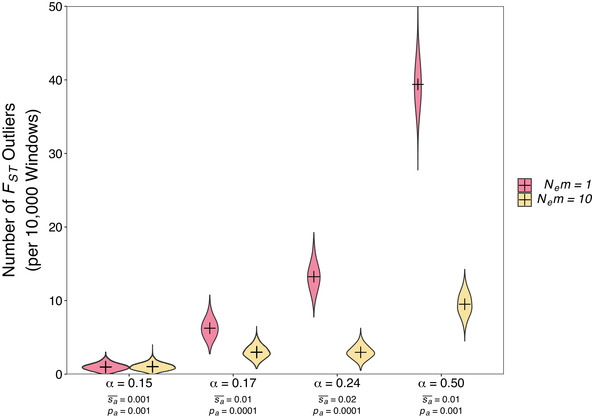
The number *F_ST_* outliers per 10,000 windows for parapatric populations subject to global adaptation. The text on the horizontal axis details the proportion of adaptive substitutions (*α*) observed in simulated populations as well as parameters of the distribution of fitness effect assumed. The mean effect of beneficial mutations is given by ${\bar{S}}_{a}$ and the proportion of new mutations that were beneficial is given by *p_a_*. *F_ST_* was calculated for 10,000 bp analysis windows centered on simulated “gene‐like regions” using the method of Weir and Cockerham (1984). Outliers were determined using the 99.99th percentile of the distribution of *F_ST_* from neutral simulations. Plusses indicate the point estimate and violins indicate the distribution of 1000 bootstraps samples from 2000 simulated datasets. Populations were simulated with *N* = 5000 diploid individuals per deme.

Figure 2 shows cases where *α* ranged from 0.15 to 0.50, where *F_ST_* outliers driven by globally beneficial mutations were often observed. For example, under parameters that most closely match what has been estimated for natural populations of *D. melanogaster* (${\bar{S}}_{a}$ = 0.02 and *p_a_* = 0.0001), we found there was an average of 13 *F_ST_* outliers for every 10,000 analysis windows in a low migration case (*N_e_m* = 1) and three outliers in a high migration case (*N_e_m* = 10). Note that the distribution of fitness effects for beneficial mutations is not well understood for natural populations and a given value of *α* can arise under various combinations of selection parameters (Supporting Information Appendix S1). For example, a high frequency of weakly beneficial mutations may yield the same adaptive substitution rate as a comparatively small number of strongly selected mutations. Indeed, Figure 2 shows cases where different distributions of beneficial fitness effects give rise to similar *α*, but that these exhibit quite different numbers of *F_ST_* outliers (compare *α* = 0.15 and *α* = 0.17 in Fig. 2). The reason for this is that weakly selected mutations take a longer time to fix and are less likely to cause elevated *F_ST_* during the course of a selective sweep (Santiago and Caballero 2005; Kim and Maruki 2011). Figure S2 shows the number of *F_ST_* outliers we observed under a variety of positive selection parameters, which corresponded to *α* values spanning from 0.02 to 0.61 (Table S1). This range of *α* values is consistent with what has been estimated for numerous eukaryotic species (Galtier 2016; Rousselle et al. 2020). The number of *F_ST_* outliers increased with both the rate and strength of advantageous mutations and decreased with the migration rate (Fig. S2).

From our simulation data, we determined whether particular *F_ST_* values were outliers based on the distribution of *F_ST_* obtained from neutral simulations. We only examined windows that straddled gene‐like regions to reduce pseudo‐replication, so we may have excluded strong outliers present in adjacent windows and thus the number of outliers reported is probably conservative. Furthermore, the results shown in Figure 2 were obtained assuming an outlier threshold based on the 99.99th percentile of values from neutral simulations. When using more or less stringent thresholds, the number of *F_ST_* outliers increased or decreased, as expected (Fig. S2). For example, when using a threshold based on the 99.9th percentile, we found 36 and 18 outliers in low and high migration cases, respectively, when ${\bar{S}}_{a}$ = 0.02 and *p_a_* = 0.0001 (Fig. S2). This lower threshold is relevant because it suggests that even when global adaptation does not generate strikingly large *F_ST_* outliers, it contributes to the genome‐wide heterogeneity in *F_ST_*, or the “spikiness” of Manhattan plots. Across parameter sets we found that around half of all outliers we identified were in regions experiencing ongoing sweeps (Fig. S2) consistent with the “lag type” of *F_ST_* outlier. The other half were not, suggesting that the “Bierne type” of *F_ST_* peak may also substantially contribute to heterogeneity in *F_ST_* across the genome.

Assuming that adaptation is mutation limited, the frequency of selective sweeps is determined in large part by the population size—the more individuals there are the greater the chance that a beneficial mutation arises. Thus, all else being equal, smaller populations will experience fewer sweeps than larger ones, so there will be less chance for global adaptation to influence the landscape of *F_ST_*. The simulation results shown in Figure 2 are from meta‐populations with 10,000 individuals (5000 per deme). Decreasing the simulated population size reduced the frequency of *F_ST_* outliers for all positive selection parameters tested (Fig. S3). For example, when the population size was 5000 individuals (2500 per deme), we observed an average of 0.25 *F_ST_* outliers for every 10,000 analysis windows analyzed when ${\bar{S}}_{a}$ = 0.02 and *p_a_* = 0.0001. When the population size was reduced to 1000 individuals (500 per deme), we found very little evidence that global adaptation impacted the landscape of *F_ST_*, as under any of the parameter combinations we tested the number of outliers observed was not greater than expected under neutrality (Fig. S3). Note, however, that when we changed population sizes in our simulation we kept absolute selection parameters constant, so rates of adaptive substitution were lower in smaller populations (Table S2). Overall, these simulations suggest that global adaptation can have a large impact on the genomic landscape of *F_ST_*, but the effect is most pronounced in large populations.

In continuously distributed populations, globally advantageous mutations spread in a wave‐like fashion (Kolmogorov et al. 1937), which can generate allele frequency clines at linked sites (Barton 2000; Barton et al. 2013). When comparing populations from different points in a continuously distributed species’ range, allele frequency clines generated by the spread of globally beneficial mutations may resemble local adaptation, in a manner similar to the two‐deme case studied above (Supporting Information Appendix S2).

## DISTINGUISHING LOCAL FROM GLOBAL ADAPTATION

Examining patterns of nucleotide diversity around *F_ST_* outliers has been proposed as a way to help to infer which kind of selective processes have occurred (Bierne 2010). When local adaptation is driven by alleles with spatially antagonistic fitness effects (“antagonistic pleiotropy”) and maintained over long periods of time, linkage disequilibrium and neutral genetic diversity can build up in genomic regions surrounding the selected locus (Petry 1983; Charlesworth et al. 1997). In structured populations, selective sweeps of globally beneficial alleles typically reduce genetic diversity in the immediate vicinity of the target of selection (Barton 2000). However, the processes that lead to the lag or Bierne types of *F_ST_* outliers may leave different profiles of genetic variability than is typically expected. The different effects that global versus local adaptation have on linked diversity may, therefore, provide a means to distinguish them.

**Figure 3:**
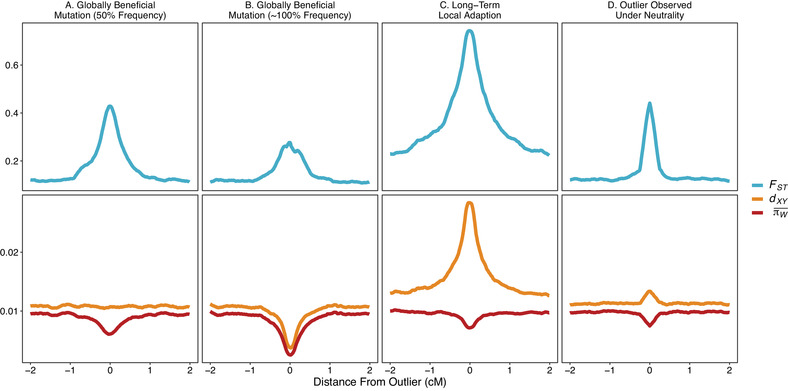
Summaries of population genomic data in regions surrounding *F_ST_* outliers generated by global adaptation, local adaptation, and genetic drift. Weir and Cockerham's *F_ST_* and nucleotide diversity between (*d_XY_*) and within ($\bar{\pi_{W}}$) populations are shown. We identified 100 *F_ST_* outliers from simulations of each of the four processes shown in the plot. Summary statistics were calculated in 10,000 bp sliding windows with a 500 bp step. In the cases of global and local adaptation, alleles with *s_a_ =* 0.05 were simulated. In all simulations shown *Nm* = 1.

We examined the patterns of *F_ST_* and nucleotide diversity within (*π_W_*) and between (*d_XY_*) populations in genomic regions surrounding *F_ST_* outliers using simulations of parapatric populations. To examine the effects of the lag‐type of *F_ST_* outlier, we sampled simulated populations when a globally beneficial mutation was at 50% frequency across the metapopulation. To examine the effects of the Bierne type, we sampled simulated populations when a globally beneficial mutation had swept to fixation (frequency greater than 99% across the metapopulation). In addition, we simulated long‐term local adaptation due to alleles with spatially antagonistic fitness effects. Figure 3 shows the average pattern of the three population genetic summary statistics in regions surrounding *F_ST_* outliers that arose in four different ways.

- Figure 3A shows that when globally beneficial mutations have spread to an intermediate frequency and generated an *F_ST_* outlier of the “lag type,” within‐population nucleotide diversity (*π_W_*) near selected sites is reduced, but between population genetic diversity (*d_XY_*) is not systematically affected.
- Figure 3B shows that the “Bierne” type of *F_ST_* outlier, that is, after a globally beneficial mutation has swept to fixation (Bierne 2010), is coincident with reductions in both *π_w_* and *d_XY_*.
- Figure 3C shows that long‐term local adaptation can result in substantially increased *d_XY_* with a slight reduction in *π_W_*.
- Figure 3D shows that even in the absence of selection, genetic drift can occasionally generate strikingly large *F_ST_*, but in such cases we found only mild perturbations in the average nucleotide diversity between or within populations.

Comparing patterns of multiple summary statistics can therefore potentially help distinguish different processes (Fig. 3). However, care should be taken, because although the mean values of *F_ST_*, *d_XY_*, and *π_W_* shown in Figure 3 may suggest quite a clear rubric for distinguishing modes of selection, the pattern of summary statistics about individual selected loci is more stochastic and potentially difficult to interpret. Figures S6–S9 show the patterns of the three summary statistics we examined around individual *F_ST_* outliers for each of the four processes shown in Figure 3.

All of the scenarios that we tested resulted in *F_ST_* peaks, but only long‐term local adaptation by spatially antagonistic alleles resulted in peaks in *d_XY_* (Sakamoto and Innan 2019). This increased *d_XY_* occurs because long‐term local adaptation acts as a local barrier to gene flow, allowing divergence to accumulate above the genomic background. Such peaks in *d*_XY_ take time to build up (e.g., Sakamoto and Innan 2019, Fig. 1), so recently established spatially antagonistic alleles may not exhibit that particular signal. Additionally, we have focused on local adaptation via spatially antagonistic alleles; if local adaptation were instead driven by conditionally beneficial alleles, these would likely only be detected using genome scans when they have recently swept to high frequencies where they are favored, but not yet spread into areas where they are neutral. Thus, conditionally beneficial alleles or recently established spatially antagonistic ones would resemble the “lag” type of *F_ST_* peak observed under global adaptation, that is, with no increase in *d_XY_*. Note that *π_W_* is not a particularly useful diagnostic for local adaptation as either increased or decreased levels relative to background can occur depending on the relative strength of migration and selection (Jasper and Yeaman 2020). Taken together, our results suggest that only prolonged periods of local adaptation driven by antagonistic pleiotropy can reliably be distinguished from global adaptation.

In our simulations of global adaptation, we observed numerous *F_ST_* outliers in regions that were not experiencing selective sweeps at the time of sampling (Fig. S2), suggesting that the process outlined by Bierne (2010) may frequently contribute to heterogeneity in *F_ST_* across genomes of natural populations. The model analyzed by Bierne (2010) predicted that there would be twin peaks of *F_ST_* about selected sites and such a profile was suggested to be diagnostic of global adaptation. We found that there was, indeed, bimodality to the distribution of *F_ST_* about selected sites once globally beneficial mutations fixed. However, for individual outliers we found that there was typically only a single peak of differentiation up‐ or downstream of the selected locus (Fig. S5). There were a few cases where global adaptation generated twin peaks of *F_ST_* about selected sites, but these were less common than single peaked profiles (Fig. S5). Twin peaked profiles of *F_ST_*, if they are present, may be diagnostic of global adaptation. However, our simulations suggest that the absence of such a pattern is also quite possible with the Bierne type of *F_ST_* outlier, so a lack of twin peaks should not be taken as evidence against global adaptation. It should be noted, however, that we examined a two‐deme model, whereas Bierne (2010) examined a one‐dimensional cline with 50 demes and patterns may be qualitatively different in those cases. Furthermore, in this study, we were concerned with structured populations connected by high levels of gene flow. The term “adaptive introgression” is used to refer to the introduction of beneficial alleles from highly diverged lineages. In cases of adaptive introgression, twin peaks of nucleotide diversity and indeed *F_ST_* about selected sites are expected (Setter et al. 2019) because of the high levels of differentiation present in the metapopulation at the onset of the sweep.

In the present study, we did not include unconditionally deleterious mutations in any of the simulations, though the patterns of summary statistics in Figure 3B qualitatively match the expectations under background selection for diverging populations (Burri 2017). For populations that undergo moderate amounts of gene flow, background selection may have little impact on *F_ST_* (Matthey‐Doret and Whitlock 2019) as deleterious mutations may migrate to other demes before being selectively eliminated, reducing both within and between population diversity to a similar extent. We focuses on cases of moderate to high gene flow, but for reviews of the signatures of selection in isolated and diverging populations, see Cruickshank and Hahn (2014); Burri (2017); and Noor and Bennett (2009).

## PATTERNS CONSISTENT WITH GLOBAL ADAPTATION IN STRUCTURED POPULATIONS ARE OBSERVED IN EMPIRICAL DATASETS

Our results, and patterns of genetic variation observed in natural populations, suggest that global adaptation should be considered along with local adaptation/divergent selection when interpreting genome scans. Figure 2 of Vijay et al., (2017) provides a close look at patterns of *d_XY_* and *π* around an *F_ST_* peak in a particular genomic region for crows, flycatchers, and Darwin's finches. For crows, they show an increase in *F_ST_* along with decreased *π*, but fairly uniform *d_XY_*, which is expected for the “lag type” of *F_ST_* outlier (Fig. 3A). For flycatchers, on the other hand, the pattern matches the expectation for an *F_ST_* outlier of the “Bierne type,” increased *F_ST_* along with both decreased *π* and *d_XY_*. The authors of that study interpreted their results in terms of a shared history of background selection, but the qualitative match with the expectations under global adaptation would seem to complicate the interpretation of the patterns. In a study of the postman butterfly (*Heliconius melpomene*), Martin et al. (2016) performed genome scans on populations from the Eastern and Western slopes of the Andes. In their study, Martin et al., (2016) found evidence for recent sweeps in the Western population that were not found in the Eastern and *vice versa*. They showed that in two cases sweep signals were coincident with increased *F_ST_* between the populations but reduced *d_XY_* and *π_W_* consistent with the process described by Bierne (2010) and similar to the patterns shown in Figure 3B. Finally, Irwin et al. (2018) examined the genomic landscape of *F_ST_*, *d_XY_*, and *π_W_* between pairs of hybridizing warbler species. They found numerous *F_ST_* peaks, many of which were coincident with reductions in *π_W_*, but not *d_XY_*, consistent with the “lag” type of *F_ST_* outlier similar to the patterns in Figure 3A. In all cases, interpretations of empirical data should be mindful of the effect of genome‐wide variation in recombination rate on all such summary statistics, as this has substantial effects on their distributions under to linked selection (Berner and Roesti 2017), and even under pure neutrality (Booker et al. 2020). When performing outlier scans for local adaptation with genomic data, patterns of multiple summary statistics should be used in combination to identify regions subject to selection. Once outliers have been identified from whole‐genome scans, data from outlier regions could be subject to fine‐scale analyses such as those proposed by Lee and Coop (2017) to determine evolutionary processes most compatible with the observations. Alternatively, simulations similar to those that we used to generate Figure 3 could be used to train machine learning‐based classifiers. Recent advances in the use of machine learning for population genomic inference suggest that this might be a fruitful avenue for further study (Flagel et al. 2019).

## GLOBAL ADAPTATION AND GENOTYPE–ENVIRONMENT ASSOCIATION STUDIES

Environments vary over space, so geographically distant populations may be subject to distinct biotic and abiotic environments. Studies may intentionally sample geographically distant populations to maximize the extent by which local adaptation shapes patterns of genetic differentiation. However, increasing the distance between sampled demes (which for species with limited dispersal is analogous to reducing the migration rate in our model of parapatry) would increase the chance that global adaptation induces *F_ST_* outliers (Supporting Information Appendix S1).

The confounding effects of global adaptation we have discussed in this perspective arise because populations may be sampled along a linear array that is parallel to a possible path taken by a globally sweeping mutation. This would be analogous to sampling along a coastline or a river valley, where a single axis determines both the spatial patterning of gene flow and environmental change. Although we did not explicitly study genotype–environment association analyses (e.g., BayEnv; Coop et al. 2010), global adaptation would also affect such studies if the environmental gradient in question had a simple spatial structure that covaried linearly with demography. The simplest solution to this problem would be to sample populations across multiple replicates of a given transition in environment, each distributed in different parts of a species range, such that it would be exceedingly unlikely that a given globally sweeping mutation would simultaneously be constricted in its spatial range to populations of a given type of environment (as per Fig. 1, Lotterhos and Whitlock 2015). Unfortunately, this will not be possible in many species if the environment of interest varies only along a single spatial dimension.

## CAVEATS: POPULATION DEMOGRAPHY, MODELS OF ADAPTATION, AND THE DISTRIBUTION OF FITNESS EFFECTS

Rates of adaptation and population demography will determine the extent to which global adaptation influences the landscape of genetic variability in natural populations. Species with large population sizes such as *D. melanogaster*, which is often estimated to have a long‐term *N_e_* of around 10^6^, likely experience frequent selective sweeps. From the coarse calculation we used in the introduction, we estimated the number of incomplete sweeps at nonsynonymous sites in *D. melanogaster* to be around 80. In a study of *D. melanogaster* populations from Zambia and Rwanda, Vy et al., (2017) found 37 loci across the genome that exhibited signatures consistent with ongoing selective sweeps. The methods used by Vy et al., (2017) were only powered to detect very strong selection (2*N_e_s_a_* > 2000), however, so alleles with more modest effects on fitness were perhaps missed by their approach. In populations with smaller sizes, global adaptation may have a limited influence on the landscape of genetic variability (Fig. S3). However, in small populations, the signal of local adaptation may also be fairly difficult to distinguish from background noise.

The average genome‐wide level of genetic differentiation is important when considering the effects of global adaptation. In our simulations, increasing the rate of gene flow decreased the frequency of *F_ST_* outliers (Fig. 2) because the probabilities of both the “lag” and “Bierne” types of *F_ST_* outlier are proportional to the migration rate (Santiago and Caballero 2005; Kim and Maruki 2011). Thus, the genomic landscape of differentiation in populations that experience high rates of gene flow will be less sensitive to the effects global adaptation than populations with more restricted gene flow. We chose migration rates of *Nm* = 1 to achieve mean *F_ST_* = 0.11 or *Nm =* 10 for mean *F_ST_ =* 0.01, as *F_ST_* in natural populations is often within this range. Of course, natural populations may exhibit more or less *F_ST_* than the cases we modeled, but our simulations results will hopefully help build intuition about the relative importance of global adaptation in the landscape of genetic differentiation.

In this perspective, we have assumed that positive selection acts on de novo mutations causing “hard” selective sweeps. However, other modes of adaptation may be frequent in nature. When positive selection acts on standing variation, for example, “soft” selective sweeps can occur (Hermisson and Pennings 2017) and analysis of population genetic data in natural populations suggests that these may be common (Garud et al. 2015; Schrider and Kern 2017). However, it is worth noting that hard sweeps in structured populations can generate profiles of summary statistics that resemble the expectations for soft sweeps in panmictic populations (Zheng and Wiehe 2019). Alternatively, multiple copies of the same beneficial allele may arise through independent mutational events, which then cause parallel selective sweeps (Ralph and Coop 2010; Ralph and Coop 2015; Paulose et al. 2019). The spread of independent copies of the same allele may cause different genetic backgrounds to hitchhike in different parts of a species’ range and generate *F_ST_* peaks about the selected locus. In cases of global adaptation by soft and/or parallel selective sweeps as we have outlined, the influence on *F_ST_* will presumably depend upon the differentiation in the region of the causal locus at the onset of selection.

Finally, we justified rates of globally beneficial mutations that we simulated based on results from the McDonald–Kreitman test and its derivatives. Such methods have been applied in estimating *α* for a variety of species (e.g., Galtier 2016; Rousselle et al. 2020). If there were no bias in estimates of *α*, there would still be uncertainty in the mode of adaptation that drove the substitutions as scenarios involving local or global adaptation may be very difficult to distinguish on the basis of nucleotide divergence. However, a major source of uncertainty, and arguably the most important for our analysis, is in the frequency of beneficial mutations and the strength of selection acting on them. The relevance of global adaptation to the genomic landscape of differentiation is tied to the frequency of strongly beneficial mutations. Similar values of *α* can arise under different distributions of fitness effects for beneficial mutations and Figure 2 shows that these may give rise to very different numbers of *F_ST_* outliers. There are currently very few estimates of the distribution of fitness effects for beneficial mutations available for natural populations, so it is difficult to make broad claims. Recent results from both humans and *Drosophila* suggest that strongly beneficial mutations may be a feature of molecular evolution (Campos et al. 2017; Uricchio et al. 2019), suggesting that global adaptation should be considered in the interpretation of genome‐scan results.

## Conclusions

Theoretical studies have demonstrated that global adaptation may influence the genomic landscape of differentiation and indeed global adaptation has been invoked to explain patterns of differentiation in natural populations before (e.g., Bierne 2010; Martin et al. 2016). What we have highlighted in this perspective is that *F_ST_* peaks driven by globally beneficial mutations are likely to be a common feature of the landscape of differentiation in large structured populations. We assumed rates of adaptation consistent with results from population genomic studies in numerous animal and plant species (e.g., Williamson et al. 2014; Galtier 2016; Hodgins et al. 2016; Rousselle et al. 2020). The *F_ST_* genome scans we implemented in this perspective are relatively simple compared the more sophisticated methods for detecting local adaptation that have been developed, for example, *FDist2, BayScan*, and *OutFlank* (Beaumont and Nichols 1996; Foll and Gaggiotti 2008; Whitlock and Lotterhos 2015). However, even such methods may be misled by global adaptation because the differentiation it can induce is highly similar to that expected under scenarios of local adaptation (Fig. 3). Patterns of genetic variability under global versus local adaptation are potentially only distinguishable using genome scans if local adaptation has been maintained for long periods of time by alleles with spatially antagonistic fitness effects. For that reason, global adaptation should be carefully considered in the design of future studies and in the interpretation of genome scan results.

## AUTHOR CONTRIBUTIONS

T.R.B, S.Y., and M.C.W. designed the study. T.R.B. performed all simulations and analyses. T.R.B. wrote the article with input from S.Y. and M.C.W.

Associate Editor: Z. Gompert

## Supporting information


**Table S1** Parameters of the DFE for advantageous mutations used in this study and the corresponding *α* values under a model of parapatry.
**Table S2** The values of α observed in under a model of parapatry.
**Figure S1** Manhattan plots of *F_ST_* calculated between parapatric populations subject to global adaptation at several time points.
**Figure S2** The proportion of analysis windows that contain *F_ST_* outliers in parapatric populations across three outlier thresholds.
**Figure S3** The proportion of analysis windows that contain *F_ST_* outliers for three differently sized parapatric populations assuming three outlier thresholds.
**Figure S4** The number of outlier SNPs in the stepping‐stone model.
**Figure S5** The pattern of summary statistics observed in the regions surrounding “Bierne type” of *F_ST_* outliers that occurred up‐ or downstream from the causal mutation.
**Figure S6** The pattern of *F_ST_*, *π_W_*, and *d_XY_* around individual *F_ST_* outliers observed when a globally beneficial mutation was at 50% frequency (i.e., the “lag type” of *F_ST_* outlier) for a two‐deme metapopulation.
**Figure S7** The pattern of *F_ST_*, *π_W_*, and *d_XY_* around individual *F_ST_* outliers observed when a globally beneficial mutation was at 100% frequency (i.e., the “Bierne type” of *F_ST_* outlier) for a two‐deme metapopulation.
**Figure S8** The pattern of *F_ST_*, *π_W_*, and *d_XY_* around individual *F_ST_* outliers observed when a spatially antagonistic allele has been segregating for 5*N_e_* generations in a two‐deme metapopulation (i.e., long‐term local adaptation).
**Figure S9** The pattern of *F_ST_*, *π_W_*, and *d_XY_* around individual *F_ST_* outliers observed under neutrality in a two‐deme metapopulation.
**Figure A1.1** The number of incomplete selective sweeps and the proportion of adaptive substitutions (α) under exponential DFEs for advantageous mutations.

## Data Availability

Analysis and plotting scripts as well as simulation configuration files are available at https://github.com/TBooker/GlobalAdaptation.
