# Supplementary material for: Global adaptation complicates the interpretation of genome scans for local adaptation
Source: Evol Lett. 2020 Dec 15;5(1):4–15. doi: 10.1002/evl3.208 (PMC7857299; doi:10.1002/evl3.208)
Supplement: Supplementary file 1 — Table S1 Parameters of the DFE for advantageous mutations used in this study and the corresponding α values under a model of parapatry. Table S2 The values of α observed in under a model of parapatry. Figure S1 Manhattan plots of FST calculated between parapatric populations subject to global adaptation at several time points. Figure S2 The proportion of analysis windows that contain FST outliers in parapatric populations across three outlier thresholds. Figure S3 The proportion of analysis windows that contain FST outliers for three differently sized parapatric populations assuming three outlier thresholds. Figure S4 The number of outlier SNPs in the stepping‐stone model. Figure S5 The pattern of summary statistics observed in the regions surrounding “Bierne type” of FST outliers that occurred up‐ or downstream from the causal mutation. Figure S6 The pattern of FST, πW, and dXY around individual FST outliers observed when a globally beneficial mutation was at 50% frequency (i.e., the “lag type” of FST outlier) for a two‐deme metapopulation. Figure S7 The pattern of FST, πW, and dXY around individual FST outliers observed when a globally beneficial mutation was at 100% frequency (i.e., the “Bierne type” of FST outlier) for a two‐deme metapopulation. Figure S8 The pattern of FST, πW, and dXY around individual FST outliers observed when a spatially antagonistic allele has been segregating for 5Ne generations in a two‐deme metapopulation (i.e., long‐term local adaptation). Figure S9 The pattern of FST, πW, and dXY around individual FST outliers observed under neutrality in a two‐deme metapopulation. Figure A1.1 The number of incomplete selective sweeps and the proportion of adaptive substitutions (α) under exponential DFEs for advantageous mutations. [file EVL3-5-4-s001.docx]

# Supplementary Material

# Methods

## Two-deme simulations

We modelled global adaptation in parapatric populations using forward-in-time simulations in *SLiM* 3.2 (Haller et al., 2019; Haller & Messer, 2019). We simulated an initial Wright-Fisher population of *N = N_e_ =* 10,000 diploid individuals (unless otherwise stated) which is later split into two equally sized demes of 5,000 individuals each. Symmetrical migration occurred between the demes with probability *m*. Simulated genomes consisted of 20 “gene-like regions” of 5,000 bp each separated by 100,000 bp of neutral sequence. Advantageous mutations occurred in the “gene-like regions” at a rate of *μp_a_* per generation and their spatially uniform fitness effects were drawn from an exponential distribution with mean $\bar{s_{a}}$. We roughly based our simulations on natural *Drosophila melanogaster* populations, where the effective mutation and recombination rates, *4Nμ* and *4Nr* respectively, are typically estimated to be around 0.01 per nucleotide per generation (Chan et al., 2012; Langley et al., 2012) . Using the tree-sequence option in *SLiM*, we recorded the coalescent histories of our simulations every 40,000 generations after the initial population split for 400,000 generations, and thus each simulation replicate gave us a total of 10 quasi-independent datasets. Neutral mutations were overlaid upon simulated genealogies at a rate of *μ* = 2.5 x 10^-7^ per bp per generation. Recombination occurred at a uniform rate of *r =* 2.5 x 10^-7^ per bp per generation. For a given set of selection and migration parameters (Table S1), we performed 200 replicate simulations. In lieu of performing extensive burn-in, we used the ‘recapitation’ feature of *PySlim*, which simulates the coalescent history of the population before the start of simulations using *msprime* (Kelleher et al., 2016). To minimise bias that may arise from grafting forward-in-time genealogies to those simulated under the coalescent, the forward component of our simulations included 1,000 generations of neutral evolution before the initial population split.

To examine the profiles of summary statistics around individual *F_ST_* outliers, we performed additional simulations to model individual globally beneficial mutations as well as local adaptation. These simulations were identical to those described above except for the following: We introduced a single copy of a selected allele in the centre of a 600 kbp chromosome 9,000 generations after the initial population split into two demes. We simulated local adaptation using a model of spatially antagonistic pleiotropy, where the selected allele conferred a heterozygotic fitness of 1 + *s_a_* in its deme of origin, but 1 / (1 + *s_a_*) in the other. We simulated the “lag type” of *F_ST_* outlier induced by global adaptation by introducing an unconditionally beneficial mutation and sampling the population when it reached a frequency of 50%. We simulated the “Bierne type” of *F_ST_* outlier by introducing an unconditionally beneficial mutation and sampling the population when it was at a frequency > 99%. Simulations where the selected allele was lost were discarded. We repeated the simulations until we had 100 *F_ST_* outliers generated by each process.

## Analysis of simulated data

From our simulation data, samples of 25 diploid individuals were drawn from each deme and used to generate VCF files. Weir & Cockerham’s (1984) estimator of *F_ST_* was then calculated for individual SNPs or from analysis windows of 10,000 base pairs using VCFtools (Danecek et al., 2011). VCFtools calculates the weighted average of *F_ST_* across sites by the average of ratios approach advocated by (Bhatia et al., 2013). For each analysis window we also calculated nucleotide diversity within populations (*π_w,i_*) and between populations *(d_XY_*).

In order to classify 10,000bp analysis windows or SNPs as outliers, we used the distribution of *F_ST_* from neutral simulations. In all aspects, the neutral simulations were identical to those modelling global adaptation, except that the *p_a_* parameter was set to 0. For each migration rate we tested (Table S1), we performed a set of 2,000 neutral simulation replicates and from them obtained the distribution of *F_ST_*, either per analysis window or per SNP. For the purpose of comparing parameter sets (Table S1), we only considered the analysis windows centred on the simulated “gene-like regions”. For each parameter set, we examined *F_ST_* across 40,000 “gene-like regions”, we used percentiles of the distribution from neutral loci as a *F_ST_* cut-offs. For example, with the 99.999^th^ percentile, we expect 0.4 outliers per parameter set in the purely neutral case. Note we also used the 99.9^th^ and 99.99^th^ percentiles as thresholds.

## Data availability

All analysis scripts and simulation configuration files are available at https://github.com/TBooker/GlobalAdaptation.

## Two-locus Stepping-Stone Simulations

We approximated the process of global adaptation in continuous space by simulating populations structured according to a one-dimensional stepping stone model. Stepping stone simulations consisted of a linear array of *k =* 500 demes arranged next to one another, with *N* = 5,000 haploid individuals per deme. Nearest-neighbour dispersal between demes occurred with probability *m* = 0.666 (0.333 in either direction). The genomes of each haploid individual consisted of two biallelic loci *A/a* and *B/b.* Individuals possessing the *a* allele had a fitness of 1 and those bearing the *A* allele had a selective advantage of 1 + *s* in all demes. The *B/b* locus was strictly neutral. Our simulations tracked the frequencies of the four possible genotypes (*AB, Ab, aB* and *ab*) through time. Recombination between the *A/a* and *B/b* loci occurred with probability *c*. Each generation, genotype frequencies were affected by the deterministic forces of selection, recombination and migration (in that order) and multinomial sampling was then used to create offspring, simulating genetic drift.

In order to achieve an equilibrium distribution of neutral allele frequencies across our simulated metapopulations, a period of burn-in was performed. However, our stepping-stone simulations consisted of 2.5 x 10^6^ individuals and in such populations, simulating the mutation and subsequent equilibration of neutrally evolving alleles would take an unfeasibly long time. To reduce simulation time, we provided a starting point for the neutral burn-in by assuming a correlation in allele frequencies between adjacent demes of *r*  = 0.99. The value of *r* we used was roughly based on results for high migration rates in the stepping-stone model given in Kimura & Weiss (1964). For a given simulation, we generated allele frequency profiles as follows. A deme was selected at random and the frequency of the *b* allele (*p_b_*) was set using a draw from a uniform distribution *U*(0, 1). Moving in both directions away from the initial deme, correlated allele frequencies were sampled using *r p_b_*(1 + *N*(0, 1 - *r*^2^)). Correlated allele frequencies were chosen until all demes have been assigned an initial value. Following the initial assignment of allele frequencies, we ran individual replicates for 100,000 generations of drift, discarding runs if the variant at the neutral locus was lost. We performed a total of 100,000 neutral simulations, recording the final allele frequency in each deme.

To simulate the spread of advantageous mutations with varying selection coefficients, we sampled a neutral simulation from the 100,000 replicates we performed and introduced a single copy of an advantageous mutation into a randomly selected deme. For all values of *s_a_* from 0.0001 to 0.1 (in increments of 0.0001), we performed 200 replicate simulations. We discarded runs where the advantageous allele was lost.

Under a given model of the distribution of fitness effects (DFE), we simulated the process of advantageous mutations occurring in our stepping-stone simulations as follows. Each generation, we draw a number of new advantageous mutations that occurred in the population as a whole, Poisson-distributed with mean *U_a_*, and assign a selection coefficient to each according to draws from the DFE. Each mutation fixes with probability *2s_a_*, and for those that do we sample a two-locus simulation with the appropriate selection coefficient. This process is repeated for 100,000 generations. At generation 99,999 we calculate *F_ST_* for neutral loci between pairs of demes using the formula for haploids from (Weir, 1994). This procedure was repeated 30 times for each combination of *U_a_* and $\bar{s_{a}}$.

**Table S1** Parameters of the DFE for advantageous mutations used in this study and the corresponding *𝛼* values under a model of parapatry. For the purposes of determining *𝛼,* a fraction 1 - *p_a_* of sites are subject to a gamma DFE for harmful mutations with shape parameter 0.3 and scale/mean 2*Ns_d_* = 200, where *s_d_* is the selection coefficient against deleterious alleles. The DFE for harmful mutations was estimated for *Drosophila melanogaster* by Loewe & Charlesworth (2006).

| ***2N***$\bar{\boldsymbol{s}_{\boldsymbol{a}}}$ | $\bar{\boldsymbol{s}_{\boldsymbol{a}}}$ | ***p_a_*** | ***𝛼*** | |
| --- | --- | --- | --- | --- |
|  |  |  | ***N_e_m* = 1** | ***N_e_m* = 10** |
| 400 | 0.02 | 0.0001 | 0.237 | 0.238 |
|  |  | 0.001 | 0.605 | 0.609 |
| 200 | 0.01 | 0.0001 | 0.165 | 0.155 |
|  |  | 0.001 | 0.503 | 0.503 |
| 100 | 0.005 | 0.0001 | 0.096 | 0.093 |
|  |  | 0.001 | 0.393 | 0.392 |
| 20 | 0.001 | 0.0001 | 0.020 | 0.020 |
|  |  | 0.001 | 0.150 | 0.151 |

*2N*$\bar{s_{a}}$ -The mean of the exponential distribution of fitness effects for advantageous mutations

*p_a_* - The proportion of new nonsynonymous mutations which are advantageous

𝛼 - The proportion of substitutions at functional sites driven by positive selection

**Table S2** The values of 𝛼 observed in under a model of parapatry. For the purposes of determining *𝛼,* a fraction 1 - *p_a_* of sites are subject to a gamma DFE for harmful mutations with shape parameter 0.3 and scale/mean 2*Ns_d_* = 200, where *s_d_* is the selection coefficient against deleterious alleles. The DFE for harmful mutations was estimated for *Drosophila melanogaster* by Loewe & Charlesworth (2006).

| $\bar{\boldsymbol{s}_{\boldsymbol{a}}}$ | ***p_a_*** | ***𝛼*** | | |
| --- | --- | --- | --- | --- |
|  |  | ***N_e_* = 1,000** | ***N_e_* = 5,000** | ***N_e_* = 5,000** |
| 0.02 | 0.0001 | 0.047 | 0.163 | 0.237 |
|  | 0.001 | 0.315 | 0.538 | 0.605 |
| 0.01 | 0.0001 | 0.023 | 0.095 | 0.165 |
|  | 0.001 | 0.186 | 0.419 | 0.503 |
| 0.005 | 0.0001 | 0.012 | 0.053 | 0.096 |
|  | 0.001 | 0.110 | 0.299 | 0.393 |
| 0.001 | 0.0001 | 0.002 | 0.012 | 0.020 |
|  | 0.001 | 0.025 | 0.089 | 0.150 |

**
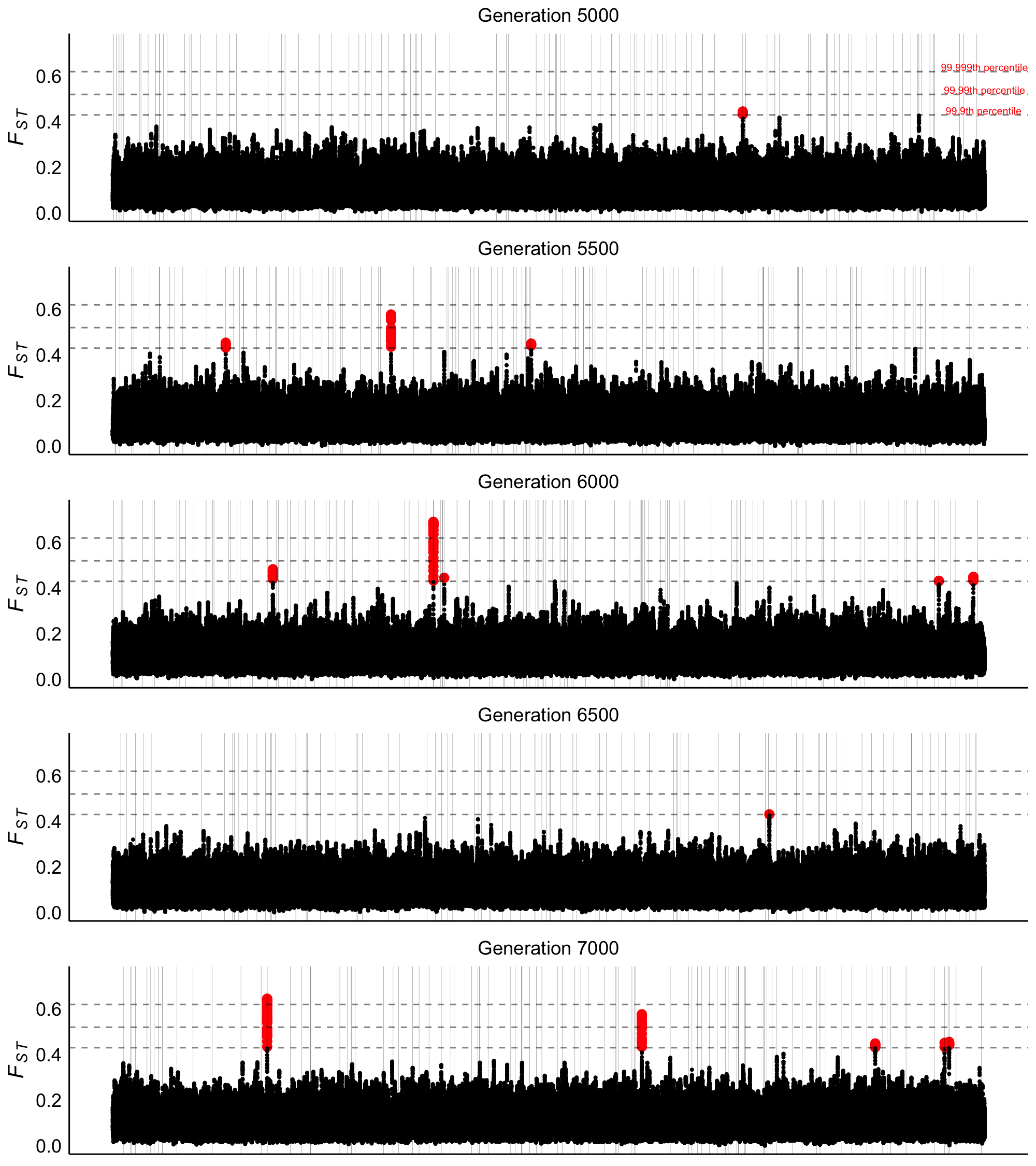
**

**Figure S1** Manhattan plots of *F_ST_* calculated between parapatric populations subject to global adaptation at several time points. *F_ST_* was calculated in sliding windows of 10,000 bp with a step size of 500 bp. The dashed horizontal lines show the 99.999^th^, 99.99^th^ and 99.9^th^ percentiles of the distribution of *F_ST_* at neutral sites as indicated on the uppermost panel. The vertical lines indicate the positions of incomplete selective sweeps. Simulation parameters, *N* = 1,000 diploid individuals per deme, *2N*$\bar{s_{a}}$ = 200, *p_a_* = 0.0001, *Nm*  = 1. The central panel, Generation = 6000, forms part of Figure 1 in the main text.

**Figure S2** The proportion of analysis windows that contain *F_ST_* outliers in parapatric populations across three outlier thresholds. Allele-frequency weighted *F_ST_* was calculated for 10,000 bp analysis windows centred on simulated “gene-like” regions. Plusses indicate the point estimate, and violins indicate the distribution of 1,000 bootstraps samples from 2,000 simulation replicates. The dashed grey horizontal line indicates the expected number of outliers under neutrality. Outlier thresholds were determined from neutral simulations.


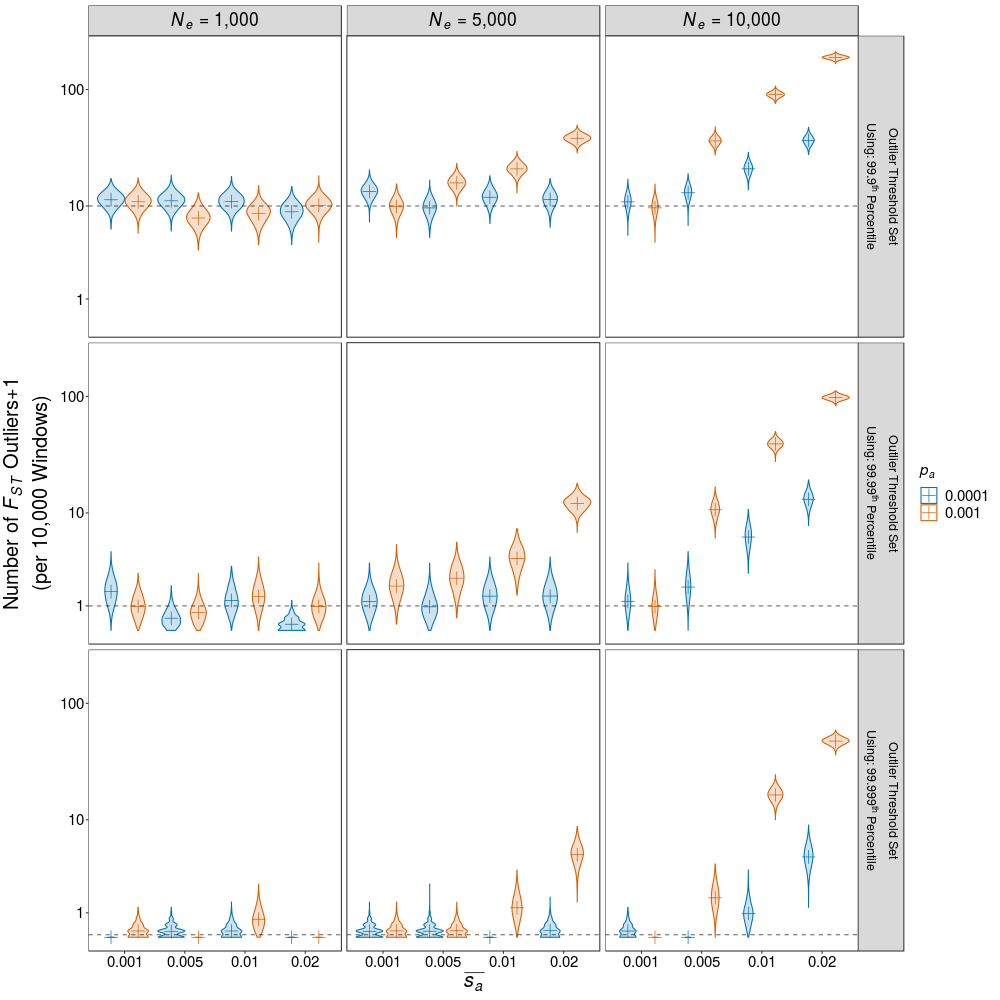


**Figure S3** The proportion of analysis windows that contain *F_ST_* outliers for three differently sized parapatric populations assuming three outlier thresholds. Allele-frequency weighted *F_ST_* was calculated for 10,000 bp analysis windows centred on simulated “gene-like” regions. Plusses indicate the point estimate, and violins indicate the distribution of 1,000 bootstraps samples from 2,000 simulation replicates. The dashed grey horizontal line indicates the expected number of outliers under neutrality. Outlier thresholds were determined from neutral simulations. Simulations assuming *N_e_m* = 1 are shown.


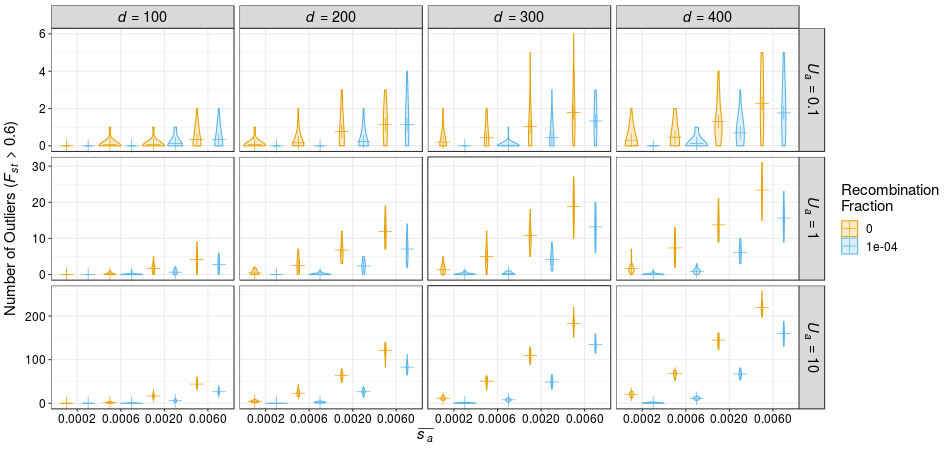


**Figure S4** The number of outlier SNPs in the stepping-stone model. The plusses indicate the mean number of outliers. Outliers are those with *F_ST_* greater than the 99.999th percentile of the distribution from neutral simulations. *U_a_* is the number of new advantageous mutations which occur each generation, *d* is the distance, in number of demes, separating the focal demes and $\bar{s_{a}}$ is the mean of an exponential distribution of advantageous mutational effects.


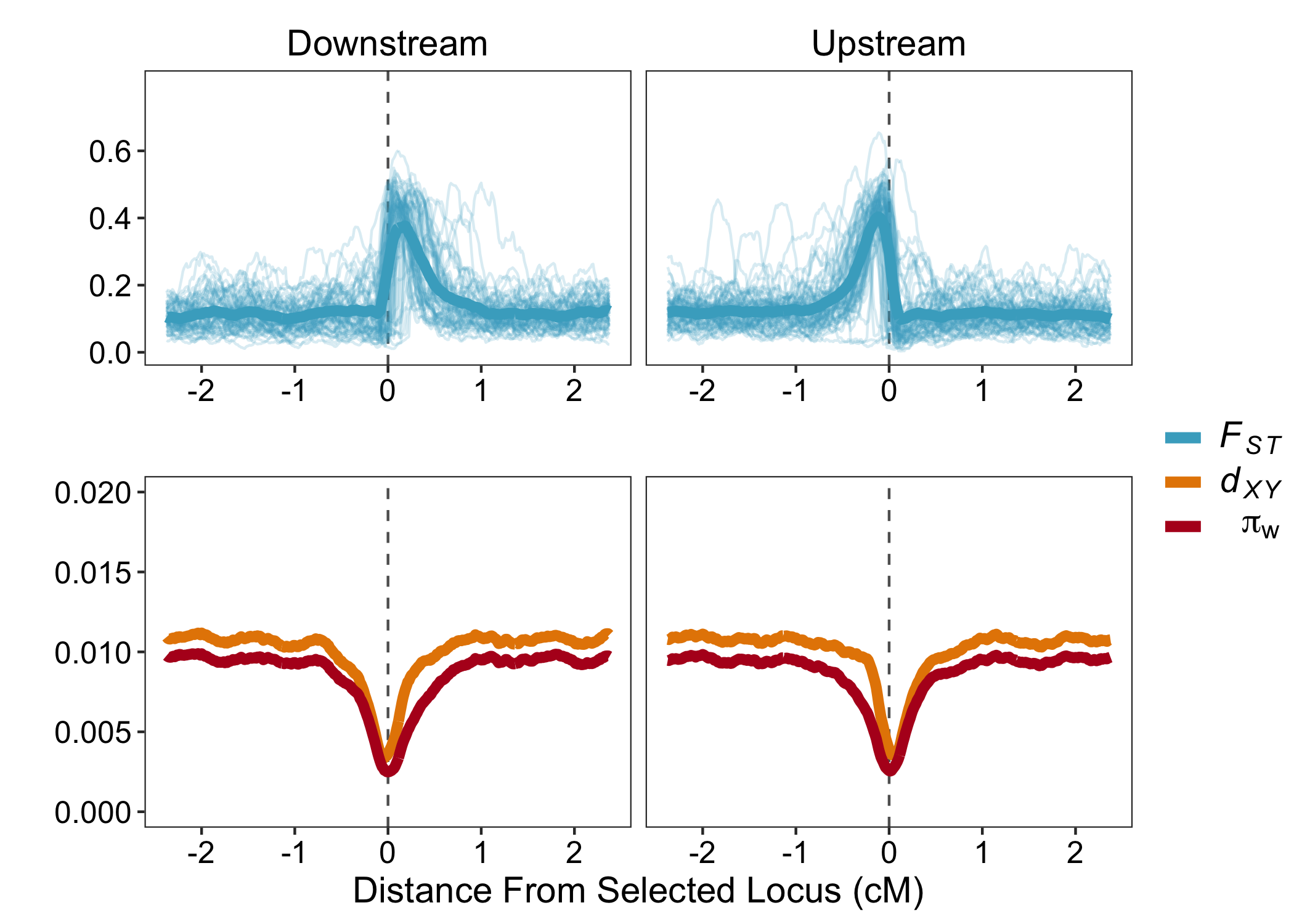


**Figure S5** The pattern of summary statistics observed in the regions surrounding “Bierne type” of *F_ST_* outliers that occured up or downstream from the causal mutation. The left-hand panels indicate cases where *F_ST_* was greatest downstream of the selected sites. The right-hand panels indicate cases where *F_ST_* was greatest upstream of the selected site. The heavy lines in each panel indicate the average profiles of *F_ST_*, *π_W_* and *d_XY_* with colours as indicated in the legend. The light lines in the upper row of plots show the pattern of *F_ST_* for individual outliers.

**Figure S6** The pattern of *F_ST_*, *π_W_*, and *d_XY_* around individual *F_ST_* outliers observed when a globally beneficial mutation was at 50% frequency (i.e. the “lag type” of *F_ST_* outlier) for a two-deme metapopulation. Each panel shows summary statistics for an independent simulation replicate. The position of the selected allele is indicated by the vertical dotted line. Summary statistics were calculated in 10,000bp sliding windows with a 500bp step. The selected allele was globally beneficial with *s_a_ =* 0.05. The migration rate was set to *N_e_m* = 1.

**Figure S7** The pattern of *F_ST_*, *π_W_*, and *d_XY_* around individual *F_ST_* outliers observed when a globally beneficial mutation was at 100% frequency (i.e. the “Bierne type” of *F_ST_* outlier) for a two-deme metapopulation. Each panel shows summary statistics for an independent simulation replicate. The position of the selected allele is indicated by the vertical dotted line. The selected allele was globally beneficial with *s_a_ =* 0.05 and the migration rate was set to *N_e_m* = 1.

**Figure S8** The pattern of *F_ST_*, *π_W_*, and *d_XY_* around individual *F_ST_* outliers observed when a spatially antagonistic allele has been segregating for 5*N_e_* generations in a two-deme metapopulation (i.e. long-term local adaptation). Each panel shows summary statistics for an independent simulation replicate.

**Figure S9** The pattern of *F_ST_*, *π_W_*, and *d_XY_* around individual *F_ST_* outliers observed under neutrality in a two-deme metapopulation. Each panel shows summary statistics for an independent simulation replicate.

# Appendix 1

## The number of ongoing selective sweeps in a panmictic populations

Developing an analytical model to accurately model the number of ongoing sweeps for in natural populations is very difficult because true underlying population structure is difficult to ascertain. Instead, we derive the number of alleles sweeping in a single panmictic population.

Consider a Wright-Fisher population of *N* diploids subject to recurrent mutations. Our model assumes free recombination, and as such we do not explicitly model deleterious variants. New mutations occur at rate *μ* per base-pair per generation. For *η_a_* sites in the genome, a proportion of new mutations generate beneficial alleles (*p_a_*). These alleles are advantageous with selective effects drawn from an exponential distribution with mean 2*N_e_*$\bar{s_{a}}$. We use the fixation time of an advantageous allele from (Ewens, 1979):

$T_{int}(N_{e},s_{a})= \int_{p_{1}}^{p_{2}} \frac{(e^{2N_{e}s_{a}x}-1)(e^{2N_{e}s_{a}(1-x)}-1)}{s_{a}x\left( 1-x \right){(e}^{2N_{e}s_{a}}-1)} dx$ Equation A1.1

To obtain an estimate of the expected time a sweeping allele spends at intermediate frequency during its sojourn, we integrate from *p_1_* to *p_2_*_._ Given a distribution of fitness effects for new mutations, there will be a distribution of expected times to fixation. To obtain the average of the distribution of times to fixation, we integrate over the distribution of fitness effects (DFE), giving:

$\hat{T}_{int}\left( N_{e},\bar{s_{a}} \right)= \int_{\frac{1}{2N_{e}}}^{\infty} \Phi\left( s_{a} \right|\bar{s_{a}})T_{int}\left( N_{e}, s_{a} \right)ds_{a}$ Equation A1.2

where Φ(*s_a_* | $\bar{s_{a}}$ ) is the probability density function of the distribution of fitness effects. The number of new advantageous mutations which arise each generation per individual, *U_a_*, is a product of the mutational target size *η* and mutation rate for advantageous alleles *μ_a_* (*μ_a_* = *μp_a_*).

$U_{a}=2\eta_{a} \mu p_{a}$ Equation A1.3

The selection coefficients and corresponding fixation probabilities for the new advantageous mutations will vary according to a distribution of fitness effects. Integrating over the DFE, we obtain the expected number of new advantageous alleles, destined for fixation, which arise each generation (*V_a_*),

$V_{a}={NU}_{a} \int_{\frac{1}{2N_{e}}}^{\infty} P_{fix}\left( s_{a}, N_{e} \right) ɸ \left( s_{a} | \bar{s_{a}} \right)ds.$ Equation A1.4

In this study we assume that the DFE for advantageous mutations is an exponential distribution with mean $\bar{s_{a}}$.

The expected number of ongoing sweeps, *X_a_* can be calculated as:

$X_{a}=\hat{T}_{int}V_{a}$ Equation A1.5

## The proportion of substitutions attributable to positive selection, *α*

If all adaptive substitutions are due to selection acting on *de novo* mutations, the proportion of substitutions attributable to positive selection (*α*) can be obtained by integrating over the relevant portions of the DFE:

$\alpha= \frac{\left( p_{a}\int_{\frac{1}{2N_{e}}}^{\infty} P_{fix}\left( s, N_{e} \right) ɸ \left( s_{a} | \bar{s_{a}} \right)ds \right)}{\left( p_{a}\int_{0}^{\infty} P_{fix}\left( s, N_{e} \right) ɸ \left( s_{a} | \bar{s_{a}} \right)ds \right)+(\left( 1-p_{a} \right)\int_{-\infty}^{0} P_{fix}\left( s, N_{e} \right) ɸ\left( s_{d} | ŝ_{d} , \beta\right)ds)}$ Equation A1.6

Where *P_fix_*(*s*) is the probability of fixation for a new mutation with selection coefficient *s*. *P_fix_* is calculated using Kimura’s formula:

$P_{fix}(s, N_{e})=\frac{(1- e^{-s})}{(1- e^{-2N_{e}s})}$

In the case of advantageous mutations with 2*N_e_s_a_* > 1, *P_fix_* is approximately *2s*. The integral in the numerator is from 1/2*N_e_* to ∞ because weakly advantageous mutations with fitness effects *2N_e_s_a_* ≤ 1 have fixation probabilities similar to neutral ones and thus it cannot be determined whether substitutions of such alleles are due to positive selection or drift.

A Mathematica workbook containing an implementation of this model is available at <https://github.com/TBooker/GlobalAdaptation>.

## The number of incomplete range-wide selective sweeps for varying 𝛼

In both parapatric and stepping-stone populations, outliers can be driven by incomplete selective sweeps (Figures S2). Fixation times for beneficial alleles are longer in structured populations than under panmixia by an amount that is inversely proportional to 1 - *F_ST_* (Whitlock, 2003). Thus, it seems reasonable to conclude that, all else being equal, the number of advantageous mutations spreading to fixation at any one time will be greater for a structured population than under panmixia. We therefore modelled the number of incomplete selective sweeps under panmixia to obtain a lower bound for the expected number in structured populations.

Under a variety of DFEs, a large number of incomplete sweeps are expected at any one time in panmictic populations (Figure A1.1A). For the same rate of beneficial mutations, the number of incomplete sweeps increases with the mean of the DFE, but asymptotes at around $\bar{s_{a}}$ ~ 0.01 in a population of 10,000 individuals (Figure A1.1A). Advantageous mutations with approximately *s_a_* > 0.01 spend a similar amount of time at intermediate frequencies. As the mean of the exponential DFE for advantageous mutations increases, a similar proportion of mutations have fitness effects *s_a_* > 0.01 and thus most mutations spend a similar amount of time spreading to fixation, which explains the asymptote in Figure A2.1A. As expected, the number of incomplete sweeps in our simulated parapatric populations exceeded the expectation under panmixia in all cases, with an average of ~70 incomplete sweeps under *p_a_* = 0.0001 and ~700 when *p_a_* = 0.001 (Figure A1.1A). Estimates of the DFE for advantageous mutations have only been obtained for a handful of organisms, but the proportion of adaptive substitutions (𝛼) has been estimated in numerous species to be around 0.5 (e.g. Galtier, 2016). Figure A1.1A shows that numerous DFEs for beneficial mutations result in many incomplete sweeps, and Figure A1.1B shows that the same DFEs give rise to *𝛼* values consistent with those that have been published. Taken together, these results suggest that the number of incomplete sweeps in natural populations may be quite large, and if that is the case, the chances of observing *F_ST_* outliers when performing studies of local adaptation is as well.

**Figure A1.1** The number of incomplete selective sweeps and the proportion of adaptive substitutions (𝛼) under exponential DFEs for advantageous mutations. A) The solid lines show the expected number of incomplete selective sweeps in panmictic populations, calculated from Equation A1.5, the crosses are from parapatric populations simulated under *Nm =* 1. B) The proportion of substitutions driven by positive selection. The solid lines were obtained using Equation A1.6. Parameters not specified in the plot, *N_e_* = 10,000, *4N_e_μ* = 0.01, *η_a_* = 2.5 x 10^7^.

# Appendix 2

## The proportion of a sweep’s duration in which it may induce differentiation between two points in a one-dimensional range.

Consider the spread of advantageous alleles in a spatially continuous, one-dimensional environment. The classical results of Fisher (1937) and Kolmogorov et al., (1937) showed that advantageous alleles spread through space in wave-like fashion at a constant speed of $v= \sigma\sqrt{2s_{a}}$, assuming a Gaussian dispersal kernel with standard deviation *σ*. Similar results have been reported for a variety of dispersal kernel (Ralph & Coop, 2010). Here, we derive a simple approximation for the proportion of the fixation time in which differentiation may be induced between two populations in a one-dimensional range.

Consider a species dispersed with uniform density over a 1-dimensional landscape of length *R*. Populations *A* and *B* are located at positions *x*(*A*) and *x*(*B*), respectively and are separated by a distance of *Δx*. Here, we derive the proportion of a sweep’s sojourn time in which it may induce genetic differentiation between regions *A* and *B.* We assume that advantageous mutations spread in wave-like fashion, and that *Δx* is much greater than the length of the wave front. Furthermore, we assume that differentiation may be induced if the wave is somewhere between regions *A* and *B*.

For any advantageous mutation occurring on the landscape that sweeps to fixation, there are two possibilities; that it occurs between *A* and *B,* which happens with probability $\frac{\Delta x}{R}$*,* or that the mutation occurs either side of the interval between *A* and *B*, this happens with a probability of $1- \frac{\Delta x}{R}$. If the beneficial mutation occurs somewhere between *A* and *B*, on average the sweep has to travel a distance of $\frac{\Delta x}{4}$ before it reaches the closest of the two populations, at which point the two populations will be genetically different at this locus. On average, the sweep has to travel $\frac{3\Delta x}{4}$ until it reaches the second population. The times that it takes for these two events to occur are $\frac{\Delta x}{4\nu}$ and $\frac{3\Delta x}{4\nu}$, respectively. Thus, on average the amount of time after the sweep has reached one population but not the other is $\frac{\Delta x}{2v}$. If the mutation lands outside of the region between *A* and *B*, the allele has to travel a distance of *Δx*, and this takes a time of $\frac{\Delta x}{v}$. The mean time an advantageous mutation may generate differentiation is thus,

$T_{diff}=\left( \frac{\Delta x}{2v} \right)\left( \frac{\Delta x}{R} \right)+ \left( \frac{1-\Delta x}{R} \right)\left( \frac{\Delta x}{v} \right)$, Equation A2.1

which reduces to,

$T_{diff}=\left( \frac{\Delta x}{v(1-\left( \frac{\Delta x}{2R} \right))} \right)$. Equation A2.2

The mean time for a complete sweep depends on the length of the species’ range. For any new mutation introduced at a random point in a species’ range, the average distance from the point of origin to the farthest range limit is $\frac{3R}{4}$. The expected fixation time is thus approximately $\frac{3R}{4v}$. The proportion of a sweep’s fixation time (*P_diff_*), in which it may induce differentiation between *A* and *B* is

$P_{diff}= \frac{T_{diff}}{T_{fix}}= \frac{\left( \frac{\Delta x}{v(1-\left( \frac{\Delta x}{2R} \right))} \right)}{\left( \frac{3R}{4v} \right)}$ , Equation A2.3

which simplifies to

$P_{diff}= \frac{\Delta x (1-\left( \frac{\Delta x}{2R} \right))}{3R}$ . Equation A2.4

Wave speed cancels out in Equation A2.4 if we assume the wave is moving at a constant speed across the landscape.

## The frequency of *F_ST_* outliers in spatially continuous populations

We approximated adaptation in continuously distributed populations by simulating a one-dimensional stepping-stone model with many demes. In these simulated populations, the number of *F_ST_* outliers at neutral sites linked to selected loci were positively related to the rate and average strength of advantageous mutations (Figure S4). When contrasting points in a continuous range, the probability of an ongoing sweep separating the two increases with physical distance (Equation A2.4), and accordingly, so did the number of *F_ST_* outliers in our simulations (Figure S4). As advantageous mutations spread through the population, recombination decouples them from linked neutral sites, decreasing the frequency of *F_ST_* outliers (Figure S4). In spatially extended populations historical population expansions may have caused allelic surfing (Klopfstein et al., 2006), resulting in an excess of high *F_ST_* regions at neutral sites (Lotterhos & Whitlock, 2015).

**References not present in the main text**

Klopfstein, S., M. Currat, and L. Excoffier 2006. The fate of mutations surfing on the wave of a range expansion. *Mol. Biol. Evol*. https://doi.org/10.1093/molbev/msj057
